# Association Between GLP1 RAs Use and Risk of Colorectal Cancer: A Systematic Review and Meta‐Analysis

**DOI:** 10.1002/hsr2.70490

**Published:** 2025-02-19

**Authors:** Ganesh Bushi, Shilpa Gaidhane, Suhas Ballal, Sanjay Kumar, Mahakshit Bhat, Shilpa Sharma, M. Ravi Kumar, Aashna Sinha, Mahalaqua Nazli Khatib, Nishant Rai, Sanjit Sah, Sorabh Lakhanpal, Muhammed Shabil

**Affiliations:** ^1^ Center for Global Health Research, Saveetha Medical College and Hospital, Saveetha Institute of Medical and Technical Sciences Saveetha University Chennai Tamil Nadu India; ^2^ Evidence for Policy and Learning, Global Center for Evidence Synthesis Chandigarh India; ^3^ One Health Centre, Jawaharlal Nehru Medical College, Datta Meghe Institute of Higher Education Wardha Maharashtra India; ^4^ Department of Chemistry and Biochemistry, School of Sciences JAIN (Deemed to be University) Bangalore Karnataka India; ^5^ Department of Allied Healthcare and Sciences Vivekananda Global University Jaipur Rajasthan India; ^6^ Department of Medicine, National Institute of Medical Sciences NIMS University Rajasthan Jaipur Rajasthan India; ^7^ Chandigarh Pharmacy College, Chandigarh Group of Colleges‐Jhanjeri Mohali Punjab India; ^8^ Department of Chemistry Raghu Engineering College Visakhapatnam Andhra Pradesh India; ^9^ Uttaranchal Institute of Pharmaceutical Sciences, Division of Research and Innovation Uttaranchal University Dehradun Uttarakhand India; ^10^ Division of Evidence Synthesis, Global Consortium of Public Health and Research, Datta Meghe Institute of Higher Education Wardha Maharashtra India; ^11^ Department of Biotechnology Graphic Era (Deemed to be University) Dehradun Uttarakhand India; ^12^ Department of Allied Sciences Graphic Era Hill University Dehradun Uttarakhand India; ^13^ SR Sanjeevani Hospital Kalyanpur Siraha Nepal; ^14^ Department of Paediatrics Dr. D. Y. Patil Medical College, Hospital and Research Centre, Dr. D. Y. Patil Vidyapeeth Pune Maharashtra India; ^15^ Department of Public Health Dentistry Dr. D.Y. Patil Dental College and Hospital, Dr. D.Y. Patil Vidyapeeth Pune Maharashtra India; ^16^ School of Pharmaceutical Sciences Lovely Professional University Phagwara Punjab India; ^17^ University Center for Research and Development, Chandigarh University Mohali Punjab India; ^18^ Medical Laboratories Techniques Department AL‐Mustaqbal University Hillah Babil Iraq

**Keywords:** colorectal cancer, glucagon‐like peptide‐1 receptor agonists, meta‐analysis, systematic review

## Abstract

**Background and Objective:**

As the global prevalence of type 2 diabetes mellitus (T2DM) continues to rise, addressing its associated health risks, including colorectal cancer (CRC), is important. This study examines the relationship between the use of glucagon‐like peptide‐1 receptor agonists (GLP‐1RAs) and the risk of CRC in comparison with other antidiabetic therapies.

**Methods:**

We conducted a systematic search of PubMed, Embase, and Web of Science up to August 10, 2024, following PRISMA guidelines. Data extraction and screening were performed using Nested Knowledge software. Meta‐analysis random effect model pooled Risk ratios (RRs) calculated using was performed using R v4.4 statistical software g. The protocol was registered with PROSPERO.

**Results:**

Out of 1825 identified studies, five met the inclusion criteria, involving 2,047,256 T2DM patients assessing CRC risk. GLP‐1RAs were associated with a significant reduction in CRC risk compared to thiazolidinediones (RR: 0.82, 95% CI: 0.68–0.96), insulin (RR: 0.57, 95% CI: 0.32–0.81), and SGLT2 inhibitors (RR: 0.77, 95% CI: 0.59–0.95). Comparisons with sulfonylureas, DPP‐4 inhibitors, and metformin were not statistically significant. A potential protective effect against alpha‐glucosidase inhibitors was observed (RR: 0.59, 95% CI: 0.18–1.00) but requires further investigation.

**Conclusion:**

The use of GLP‐1RAs in T2DM is linked to a reduced risk of CRC compared to several standard antidiabetic therapies. These findings underscore the importance of considering long‐term cancer risks in diabetes management and highlight the need for continued research to fully understand the implications of GLP‐1RA use in T2DM patients.

## 1 Introduction

The escalating global prevalence of type 2 diabetes mellitus (T2DM) poses significant challenges to healthcare systems worldwide. As of 2021, more than 537 million adults are affected, with projections suggesting an increase to over 783 million by 2045 [1]. Managing diabetes and its associated complications requires effective therapeutic strategies. In this context, glucagon‐like peptide‐1 receptor agonists (GLP‐1 RAs) represent a significant advancement in the pharmacological treatment of T2DM [2]. Initially approved for glycemic control, GLP‐1 RAs have also shown benefits in weight loss, cardiovascular protection, and potentially anti‐inflammatory properties [3]. However, their expanding use has raised concerns about long‐term safety, particularly regarding the risk of malignancies such as colorectal cancer (CRC), which ranks third in incidence and second in mortality among cancers globally [4].

The relationship between T2DM and CRC has been thoroughly investigated, revealing that individuals with diabetes are at an increased risk of developing CRC [5]. This association is thought to be mediated through several pathophysiological mechanisms, including hyperinsulinemia, chronic inflammation, and changes in gut microbiota [6]. With the introduction of GLP‐1 RAs into clinical practice, there has been increased scrutiny of their impact on CRC risk due to their effects on glucose metabolism and gastrointestinal physiology [7].

In managing T2DM, GLP‐1RAs are broadly recognized for their efficacy. These drugs are primarily synthetic or semi‐synthetic analogs of the human incretin hormone GLP‐1, designed to mimic its glucose‐lowering effects [8]. Some, like exenatide, are derived from exendin‐4, a peptide from the Gila monster's saliva, which, while structurally similar to human GLP‐1, offers distinct pharmacokinetic properties [9]. Other GLP‐1RAs, such as liraglutide, semaglutide, and dulaglutide, are modified forms of human GLP‐1 with structural alterations to enhance stability, extend half‐life, and improve therapeutic outcomes [10]. Despite their therapeutic benefits, there is concern over their safety profile, especially their potential role in cancer development [11]. Preclinical studies on GLP‐1 RAs have shown varying effects on cell proliferation and apoptosis, including in the colon, with some studies suggesting an increased risk of colorectal neoplasms and others indicating potential protective effects [12, 13, 14].

This systematic review and meta‐analysis aims to synthesize the existing evidence on the association between GLP‐1 RA use and the risk of CRC. By pooling data from studies, this analysis seeks to provide a comprehensive understanding of the implications of GLP‐1 RA use in clinical practice.

## 2 Methods

This review adhered to the PRISMA guidelines to ensure methodological rigor and transparency [15] (Table S1) The protocol was registered in PROSPERO (CRD42024577765).

### 2.1 Data Source and Search

A comprehensive search of electronic databases, including PubMed, Embase, and Web of Science, was conducted to identify relevant studies. The search covered all literature from the inception of each database until July 10, 2024. Keywords and Medical Subject Headings (MeSH) related to “GLP‐1 receptor agonists,” “colorectal cancer,” “neoplasms,” and “type 2 diabetes” were used to formulate the search strategy. Additionally, reference lists of all included studies, and relevant review articles were manually examined to identify any additional studies that met the inclusion criteria. The search process was not restricted by language to ensure a comprehensive collection of relevant data (Table S2).

### 2.2 Screening and Data Extraction

Two independent reviewers conducted a preliminary screening of titles and abstracts to identify potentially relevant studies using Nested Knowledge software. Full texts of articles that appeared to meet the inclusion criteria were retrieved for detailed assessment. Studies were included if they involved adults aged 18 years or older with T2DM and focused on the use of GLP‐1 receptor agonists (e.g., exenatide, liraglutide, and semaglutide), specifically reporting on the incidence of CRC Eligible study designs included cohort and case‐control studies. Studies involving patients with type 1 diabetes, those not reporting CRC as an outcome, non‐original research articles, and animal studies were excluded. Any discrepancies in study selection between the two reviewers were resolved through discussion or consultation with a third reviewer.

Data extraction was conducted independently by two reviewers using the Nested Knowledge software. The extracted data encompassed study characteristics such as author(s), year of publication, country, study design, and sample size. Participant characteristics, details about the intervention, were extracted alongside information about the comparison group. The primary outcome of interest was the incidence of CRC, documented including any reported risk estimates such as odds ratios or hazard ratios with corresponding 95% confidence intervals.

### 2.3 Quality Assessment

The quality of the included studies was independently assessed by two reviewers using validated tools appropriate for the study design. The Newcastle‐Ottawa Scale (NOS) was used for cohort and case‐control studies. Each study was rated based on predefined criteria to determine whether it posed a low, moderate, or high risk of bias. Any discrepancies in the quality assessment were discussed and resolved, with a third reviewer available for consultation if necessary.

### 2.4 Evidence Synthesis

Statistical analyses were conducted using R v.4.4 statistical software. The primary analysis involved calculating pooled estimates of the association between GLP‐1 receptor agonist use and the risk of CRC. Risk ratios (RRs) and their 95% confidence intervals (CIs) were used to evaluate this association. For studies presenting hazard ratios (HRs), these were directly interpreted as RRs. In cases where studies reported odds ratios (ORs), the data were transformed into RRs for inclusion in the meta‐analysis using the formula: RR = OR/([1 − pRef] + [pRef × OR]), where pRef represents the prevalence of CRC in the reference group [16, 17]. This method ensures that all measures of association are consistently analyzed as RRs, facilitating a coherent synthesis of the data. Heterogeneity among the included studies was assessed using the I² statistic, with values exceeding 50% indicating substantial heterogeneity [18]. The potential for publication bias was evaluated using funnel plots and Egger's test for asymmetry if a minimum of 10 studies were available. If evidence of publication bias was detected, the trim‐and‐fill method was applied to adjust the pooled estimates accordingly, providing a more accurate reflection of the association between GLP‐1 receptor agonist use and CRC risk [19].

The results of the systematic review and meta‐analysis were reported following PRISMA guidelines. A flow diagram illustrated the process of study selection, while tables summarized the characteristics of the included studies and the findings of the meta‐analysis. Forest plots visually presented the effect estimates from individual studies as well as the pooled estimates derived from the meta‐analysis.

## 3 Results

### 3.1 Literature Search

**FIGURE 1:**
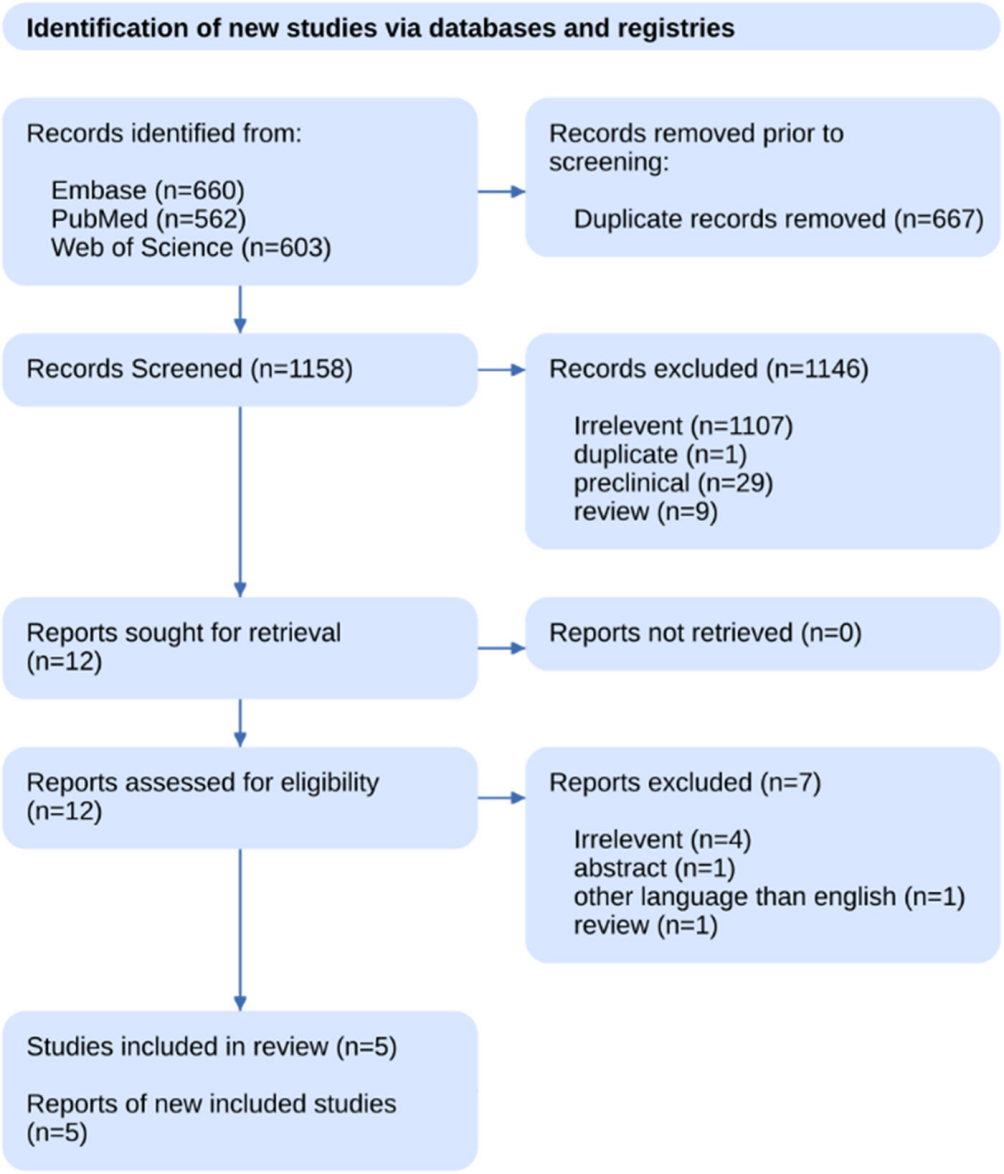
PRISMA flow chart.

Initially, a total of 1825 studies were identified through comprehensive searches across multiple databases: Embase (660 studies), PubMed (562 studies), and Web of Science (603 studies) (Figure 1). Duplicate entries were then removed, totaling 667, leading to 1158 studies being screened. After screening, a substantial number of studies were excluded (1146 studies), primarily due to irrelevance (1107 studies), along with exclusions due to duplication (1 study), preclinical nature (29 studies), and review articles (9 studies). Consequently, 12 studies were selected for full‐text retrieval and further assessed for eligibility. Of these, 7 reports were excluded due to various reasons: irrelevance (4 studies), being an abstract only (1 study), non‐English language (1 study), and being a review article (1 study). Ultimately, five studies met the eligibility criteria and were included in the systematic review. This flowchart exemplifies a structured and rigorous approach to literature screening and selection, ensuring that the systematic review is based on relevant and high‐quality studies.

### 3.2 Characteristics of Studies Included

In this analysis, five studies were included, encompassing a total sample size of approximately 2,047,256 individuals from diverse geographical regions, such as Canada, the United States, and the United Kingdom (Table 1). These studies utilized both prospective and retrospective cohort designs and examined a wide age range, starting at 40 years. The analyses primarily assessed the efficacy of GLP‐1RAs in comparison with various antidiabetic treatments, including thiazolidinediones, sulfonylureas, sodium‐glucose cotransporter‐2 inhibitors, dipeptidyl‐peptidase 4 inhibitors, alpha‐glucosidase inhibitors, metformin, and insulin. The studies' quality, assessed using the NOS, ranged from moderate to high (Table S3).

**TABLE 1:** Summary characteristics so the studies included.

Study	Age (mean years)	Country	Male %	Study design	Ethnicity	BMI (kg/m^2^)	No. of smokers	Treatment duration	Total sample size	Effect size (OR, HR, RR, 95% CI)	Comparison groups	Population	Duration of therapy	Key findings
Wang, Lindsey 2024 [20]	NA	Canada	NA	Prospective Cohort	Hispanic/Latinx‐ 8.7% Not Hispanic/Latinx‐ 65.1 Unknown‐ 26.3	NA	NA	15 years follow up	1,221,218	HR (GLP‐1RAs versus TZD) = 0.82 (0.69–0.97); versus SU = 0.82 (0.68–0.98); versus SGLT2 = 0.77 (0.62–0.97); versus DPP‐4 = 0.93 (0.78–1.10); versus AGI = 0.59 (0.31–1.13); versus Metformin = 0.75 (0.58‐0.97); versus Insulin = 0.56 (0.44–0.72)	GLP‐1RAs versus TZD, SU, DPP‐4, AGI, Insulin, Metformin, SGLT2	Patients diagnosed with T2DM	15 years	GLP‐1RAs were associated with reduced CRC risk in drug‐naive T2D patients, with stronger effects in those with obesity. Some limitations include potential confounding and observational study biases.
Wang, jiasheng 2022 [21]	59.8	Cleveland, USA	50.1%	Retrospective cohort	Hispanic/Latinx‐ 8.5% Not Hispanic/Latinx‐ 67.8 Unknown‐ 23.7	Overweight or obesity: 37.1% Obesity due to excess calories: 18.4% Unspecified obesity: 27.8% Morbid obesity with alveolar hyperventilation: 0.3% BMI 30–39 (adults): 8.3% BMI ≥ 40 (adults): 7.8%	NA	15 year follow up	GLP‐1RAs versus Insulin: 48,443; GLP‐1RAs versus Metformin: 32,275	HR (GLP‐1RAs versus Metformin) = 0.88 (0.73–1.04); HR (GLP‐1RAs versus Insulin) = 0.54 (0.46–0.64)	GLP‐1RAs versus Insulin, GLP‐1RAs versus Metformin	Type 2 Diabetes, with and without obesity	March 2005 to November 2018	GLP‐1RAs were linked to a lower risk of 10 out of 13 obesity‐associated cancers (OACs) compared to insulin, suggesting a potential preventive effect against cancer.
Abrahami, Devin 2018 [22]	≥ 40	UK	57%	Prospective cohort	NA	< 25 KG/m^2^ = 1% 25–30 kg/m^2^ = 7% ≥ 30 kg/m^2^ = 60%	12%	January 1988 to March 2015	19,690	HR (GLP‐1RAs versus SU) = 1.1 (0.5–2.3)	GLP‐1RAs versus SU	Patients diagnosed with T2DM	Mean of 3.5 years	GLP‐1RAs were not associated with a substantial increase in CRC risk compared to sulfonylureas, indicating a neutral effect on CRC incidence.
Wang, Jiasheng 2022 [23]	NA	New York, USA	50.21%	Retrospective Cohort	White = 77.2% African American = 17.6 Hispanic = 1.4 Asian = 0.8	< 25 = 0.4% 25–30 = 15.3% 30–35 = 29.3% > 35 = 55%	16%	January 2005 to June 2019	683,570	OR (Exenatide versus Metformin) = 0.80 (0.66–0.97); OR (Liraglutide versus Metformin) = 0.62 (0.52–0.75); OR (GLP‐1RAs versus Metformin) = 0.72 (0.64–0.83); OR (GLP versus SU) = 0.55 (0.49–0.62)	GLP‐1RAs versus Metformin, GLP‐1RAs versus SU	Patients diagnosed with T2DM	Up to 5 years	GLP‐1RAs were associated with a lower risk of prostate, lung, and colon cancer but with a higher risk of thyroid cancer.
Htoo, Phyo T 2016 [24]	> 66	USA	40.16%	Cohort study	White = 87.4 Black = 6.1 Other races = 6.5	NA	NA	2007–2013	70,503	OR = 0.82 (0.42–1.58)	GLP‐1RAs versus Insulin	Patients diagnosed with T2DM	2007–2013	No significant effect of GLP‐1RAs on short‐term CRC risk was observed, suggesting a neutral impact.

Abbreviations: AGI, alpha‐glucosidase inhibitors; CI, confidence interval; CRC, colorectal cancer; DPP‐4, dipeptidyl peptidase‐4 inhibitors; HR, hazard ratio; NA, not available; OACs, obesity‐associated cancers; OR, odds ratio; RR, risk ratio; T2DM, type 2 diabetes mellitus; SU, Sulfonylureas; SGLT2, Sodium‐Glucose Cotransporter‐2 Inhibitors; TZD, thiazolidinediones.

### 3.3 Meta‐Analysis

**FIGURE 2:**
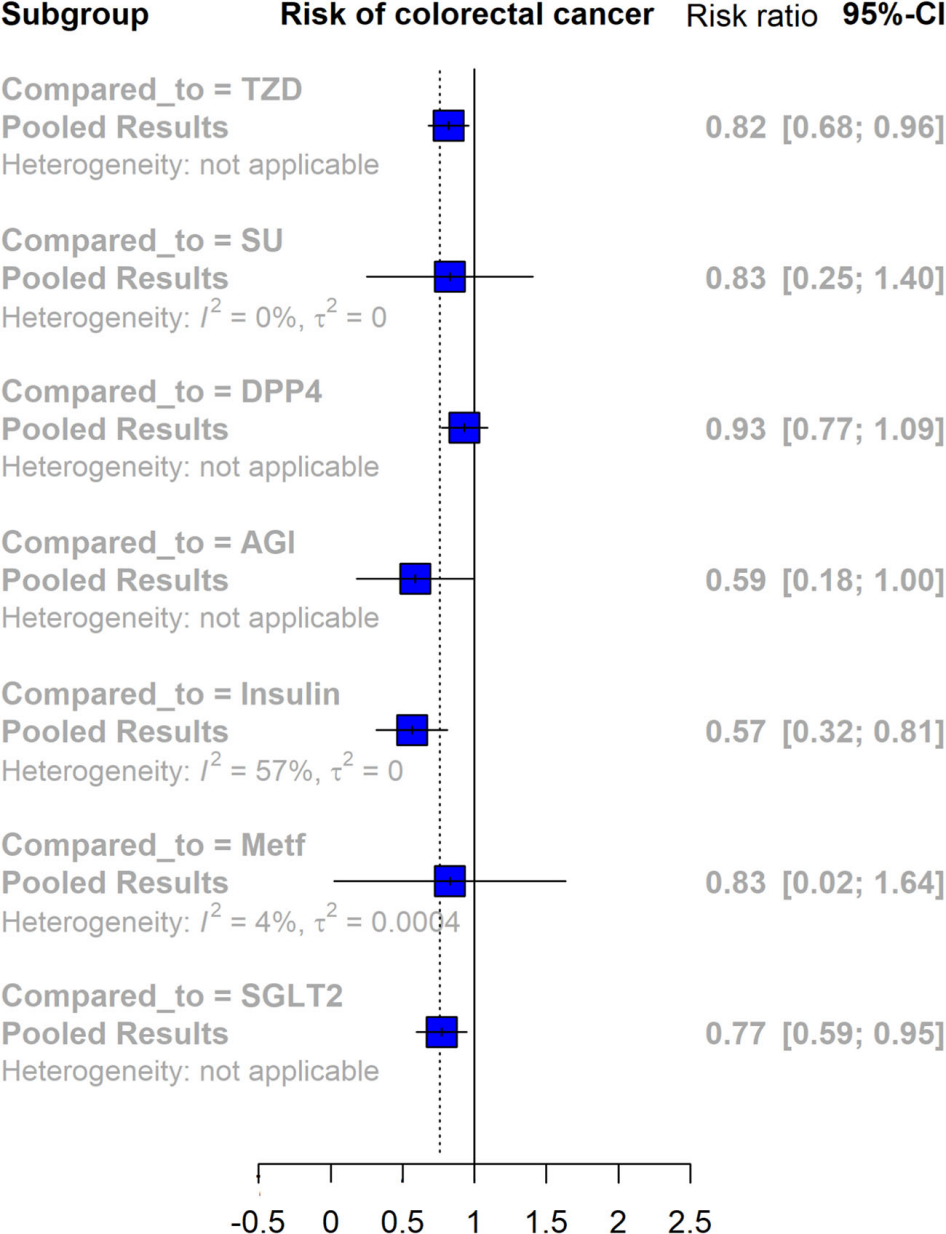
Forest plot depicting the risk of colorectal cancer associated with GLP‐1RAs use compared to other antidiabetic medications (AGI, alpha‐glucosidase inhibitors; CI, confidence interval; DPP4, dipeptidyl peptidase‐4 inhibitors; Mett, metformin; SU, sulfonylureas; SGLT2, sodium‐glucose cotransporter‐2 inhibitors TZD, thiazolidinediones).

This meta‐analysis investigates the risk of CRC associated with the use of GLP‐1RAs compared to various antidiabetic agents in patients with type 2 diabetes (Figure 2). The findings indicate a statistically significant reduction in cancer risk when comparing GLP‐1RAs to TZD, with a RR of 0.82 [95% CI: 0.68, 0.96], and to insulin, with a RR of 0.57 [95% CI: 0.32, 0.81]. The comparison with SGLT2 also shows a significant reduction, with a RR of 0.77 [95% CI: 0.59, 0.95]. In contrast, comparisons with SU, DPP4, and metformin yield nonsignificant results, indicating no clear evidence of risk reduction. The analysis of AGI suggests a potential protective effect with a RR of 0.59 [95% CI: 0.18, 1.00], though the confidence interval reaches unity, necessitating further investigation. Overall, these results suggest that GLP‐1RAs might be associated with a decreased risk of CRC compared to several traditional antidiabetic treatments, with significant findings particularly noted against insulin, TZD, and SGLT2 inhibitors.

### 3.4 Publication Bias

Due to the limited number of studies included in this analysis, it was not feasible to assess publication bias using a funnel plot or Egger's test effectively.

## 4 Discussion

This systematic review and meta‐analysis evaluated the association between GLP‐1RA use and the risk of CRC in patients with T2DM. By incorporating data from approximately 2,047,256 patients across multiple studies, our findings suggest a potential reduction in CRC risk associated with GLP‐1RAs when compared with traditional diabetes therapies such as insulin and TZDs. The results indicate an 18% reduction in CRC risk when comparing GLP‐1RAs with TZDs and a 43% reduction when compared with insulin. These findings suggest significant variation in CRC risk modulation among different antidiabetic therapies, with important implications for clinical decision‐making.

The protective effects of GLP‐1RAs may be attributed to their intrinsic pharmacological properties, including weight loss and anti‐inflammatory mechanisms, both of which are recognized as factors in reducing cancer risk [8]. Additionally, GLP‐1RAs have been shown to directly modulate cellular processes, such as proliferation and apoptosis, particularly in colonic epithelium, which may mitigate CRC development. These findings align with earlier studies highlighting the potential of GLP‐1RAs to influence tumorigenesis through these mechanisms [25].

In contrast, comparisons between GLP‐1RAs and sulfonylureas, dipeptidyl peptidase‐4 inhibitors (DPP‐4i), and metformin did not show significant reductions in CRC risk. These discrepancies underscore the complexity of diabetes pharmacotherapy and its varied implications for cancer risk [20]. Metformin, for instance, exhibits anti‐inflammatory and anti‐neoplastic properties, primarily through activation of AMP‐activated protein kinase (AMPK), which reduces cancer cell proliferation and inflammatory pathways [26]. Although the risk reduction observed with metformin was modest compared with GLP‐1RAs, its overall risk profile supports its continued use in managing T2DM, particularly given its additional metabolic benefits [27].

Systematic reviews and meta‐analyses have quantified the increased CRC risk associated with insulin and TZDs, therapies often linked to weight gain and hyperinsulinemia. For instance, insulin therapy has been associated with a RR of 1.69 (95% CI, 1.25–2.27), likely due to hyperinsulinemia‐induced cell proliferation and reduced apoptosis [28]. TZDs, such as pioglitazone, have also been implicated in cancer risk, with studies demonstrating a dose‐dependent relationship between cumulative exposure and bladder cancer incidence [29, 30]. This finding underscores the importance of considering cancer risk in selecting antidiabetic therapies, particularly for patients at higher baseline risk of malignancy.

Recent evidence also suggests the potential benefits of sodium‐glucose cotransporter‐2 inhibitors (SGLT‐2i) in cancer risk reduction. A study by Chiang et al. (2024) found that SGLT‐2i use was associated with improved 5‐year overall survival and progression‐free survival in T2DM patients with CRC, with a 50%–70% reduction in all‐cause mortality and disease progression compared with non‐SGLT‐2i users [31]. Similarly, Suzuki et al. (2024) reported an overall reduction in cancer risk (HR, 0.80) and a specific reduction in CRC risk (HR, 0.71) with SGLT‐2i [32]. These findings suggest that SGLT‐2i may offer dual benefits in glucose control and cancer prevention, warranting further investigation into their potential roles in therapeutic strategies.

The safety profile of GLP‐1RAs warrants ongoing surveillance. Although no definitive association between GLP‐1RA use and neoplasms has been established, some studies have raised concerns. A study by Funch et al. (2018) reported breast cancer and metastatic intestinal adenocarcinoma cases in patients receiving liraglutide, although these events occurred late in the study period and were not definitively linked to the drug [33]. Additionally, thyroid cell tumors observed in animal studies have not been confirmed in humans, with recent reviews suggesting no significant risk with short‐term use. Nonetheless, long‐term data remain limited, and further studies are required to assess the potential carcinogenic effects of GLP‐1RAs [34].

Gastrointestinal side effects, including nausea and diarrhea, lead to discontinuation rates as high as 10.3% for semaglutide and 8.3% for liraglutide [35]. Beyond these common adverse events, GLP‐1 RAs have been linked to more serious conditions such as pancreatitis, with a risk reportedly nine times higher than that associated with older weight‐loss medications like bupropion, although the absolute risk remains below 1% annually [36]. Additionally, a meta‐analysis of 76 randomized clinical trials with over 100,000 participants revealed an increased risk of gallbladder and biliary diseases, especially at higher doses and longer durations of use [37]. Research also shows that GLP‐1 can suppress cholecystokinin secretion, which may impair gallbladder motility and explain the increased incidence of gallbladder‐related adverse effects [38]. Other gastrointestinal issues linked to GLP‐1RAs include gastroparesis, which presents as nausea and vomiting due to delayed gastric emptying, and intestinal obstruction, occurring at a rate four times higher than in non‐users [36]. Symptoms such as abdominal pain, constipation, and severe vomiting require vigilant monitoring and comprehensive patient education on the signs of serious complications, including severe abdominal pain, inability to pass gas or move bowels, and jaundice [39, 40]. In clinical practice, it is essential to weigh these risks against the benefits, particularly for patients prone to gastrointestinal issues, and to ensure thorough patient education on recognizing symptoms of serious complications like severe abdominal pain and jaundice.

Several limitations of this study should be acknowledged. The conversion of odds ratios to risk ratios for uniformity across studies may introduce assumptions that do not universally apply, potentially biasing the results. Heterogeneity among studies, including differences in study designs, populations, treatment durations, and comparator drugs, poses challenges in drawing definitive conclusions. Moreover, the retrospective nature of included studies limits the ability to establish causality due to potential residual confounding.

The predominance of data from high‐income countries also limits the generalizability of findings to low‐ and middle‐income settings, where differences in healthcare access, baseline cancer risk, and antidiabetic medication use may influence outcomes. These gaps underscore the need for more diverse and inclusive research to validate these findings globally.

The increased CRC risk observed with insulin and TZDs may be partly attributed to insulin resistance and hyperinsulinemia, which are common in patients requiring these therapies. Both conditions are associated with increased cell proliferation and inhibition of apoptosis, mechanisms known to promote tumorigenesis. This hypothesis is supported by studies demonstrating elevated CRC risk in insulin‐treated patients with higher baseline insulin resistance. In contrast, GLP‐1RAs' potential to reduce CRC risk may reflect their weight‐lowering and anti‐inflammatory properties, as well as direct modulation of cellular processes involved in tumor development [41]. These mechanisms suggest that GLP‐1RAs offer a favorable profile for diabetes management in patients at higher risk of malignancy. However, the absence of significant CRC risk reduction compared with metformin and other glucose‐lowering agents suggests that the benefits of GLP‐1RAs may not be universal and require further investigation [20].

The potential benefits of GLP‐1RAs in glucose control and cancer prevention highlight a critical opportunity for optimizing antidiabetic therapy. Given the high burden of both T2DM and cancer, integrating these agents into therapeutic strategies could have profound implications for improving patient outcomes. However, their use must be balanced against safety considerations, particularly in patients with predisposing factors for adverse events.

Future research should focus on conducting long‐term, randomized controlled trials specifically designed to evaluate cancer outcomes as primary endpoints. Subgroup analyses considering patient demographics, duration of diabetes, and comorbidities would provide deeper insights into the mechanisms underlying the observed protective effects of GLP‐1RAs. Additionally, exploring interactions between GLP‐1RA use and lifestyle factors, such as diet and physical activity, may inform holistic approaches to diabetes management that optimize both metabolic and cancer‐related outcomes.

## 5 Conclusions

This systematic review and meta‐analysis indicate that GLP‐1RAs use in patients with T2DM is linked to a reduced risk of CRC compared to several traditional antidiabetic therapies. This finding adds a critical dimension to diabetes management, highlighting GLP‐1RAs' potential role not only in glycemic control but also in reducing cancer risk—a serious complication of diabetes. Our study emphasizes the importance of considering long‐term health outcomes in diabetes treatment and underscores the need for continued research to fully grasp the long‐term effects of GLP‐1RA use. By exploring the link between diabetes therapies and cancer risk, this work contributes to more comprehensive and patient‐centered care strategies for individuals with T2DM.

## Author Contributions

**Ganesh Bushi:** conceptualization, methodology, software, data curation, writing – review and editing. **Shilpa Gaidhane:** investigation, methodology, formal analysis, supervision. **Suhas Ballal:** writing – original draft, writing – review and editing, resources. **Sanjay Kumar:** writing – review and editing, validation, formal analysis, data curation. **Mahakshit Bhat:** conceptualization, writing–original draft, validation, visualization, software. **Shilpa Sharma:** software, visualization, writing – review and editing, writing – original draft. **M Ravi Kumar:** writing–original draft, validation, data curation. **Aashna Sinha:** validation, investigation, supervision, resources. **Mahalaqua Nazli Khatib:** validation, visualization, project administration, resources. **Nishant Rai:** writing – review and editing, formal analysis, project administration. **Sanjit Sah:** data curation, writing – review and editing, writing–original draft, investigation. **Sorabh Lakhanpal:** validation, conceptualization, writing – original draft, software. **Muhammed Shabil:** methodology, resources, writing – review and editing, writing – original draft.

## Conflicts of Interest

The authors declare no conflicts of interest.

## Ethics Statement

The authors have nothing to report.

## Transparency Statement

The lead author Mahalaqua Nazli Khatib, Sanjit Sah affirms that this manuscript is an honest, accurate, and transparent account of the study being reported; that no important aspects of the study have been omitted; and that any discrepancies from the study as planned (and, if relevant, registered) have been explained.

## Supporting information

Supporting information.

## Data Availability

The data that supports the findings of this study are available in the Supporting material of this article. The data is with the authors and available upon request.
